# Comparative and functional anatomy of masticatory muscles and bite force in opossums (Didelphimorphia, Didelphidae)

**DOI:** 10.1002/ar.25675

**Published:** 2025-04-25

**Authors:** Juann A. F. H. Abreu, Diego Astúa

**Affiliations:** ^1^ Laboratório de Mastozoologia, Departamento de Zoologia Centro de Biociências, Universidade Federal de Pernambuco Recife Brazil; ^2^ Programa de Pós‐graduação em Biologia Animal Universidade Federal de Pernambuco Recife Brazil

**Keywords:** Didelphidae, marsupial, mastication, myology, opossum

## Abstract

Opossums (Didelphidae) are American marsupials traditionally known for their generalized morphology and generalist feeding habits. They include a diversity of similar items in their diets, but the proportion of types of items consumed varies between taxa. Thus, feeding ecology shows varying degrees of omnivory or food preference that cannot be distinguished into strict dietary categories. With few exceptions, the anatomical and functional relationship between the masticatory muscles and variation in food resources used in opossums is unknown. Here we provide comparative descriptions of the jaw adductor muscle anatomy and architecture of nine Didelphidae genera. The muscles were dissected, weighed, and chemically digested for separation and measurement of the muscle fascicles. We estimated the potential physiological cross‐sectional area (pPCSA) of the muscles and used 2D lever arm mechanics to calculate the potential bite force on the canine and first molar. We tested the allometric relationships of muscle variables and bite forces and the correlation of bite forces with diet and diet mechanical challenge (relative frequency of hard items). The adductor muscles were represented by the *m. temporalis* and *m. masseter*, with two layers (superficial and deep) each, and *m. pterygoideus medialis* across the sample. The *m. zygomaticomandibularis* was also identified in most genera, except for *Didelphis* and *Lutreolina*. Muscle anatomy is conserved but varies in the extent of the attachment areas, in part due to differences in skull morphology. The anatomical diversification and relationships between muscles corresponded to a generalized pattern in most genera, which proved to be efficient for adding different items to the diet. The mass, average fascicle length, and pPCSA of the adductor muscles scaled isometrically with size. Bite forces on the canine and first molar also scaled isometrically and were not correlated with diet or diet mechanical challenge. Therefore, the variation in quantitative myological data and bite force was consistent with size, and the increase in bite force supports dietary diversification associated with increased size in opossums.

## Graphical abstract

We describe the functional anatomy of masticatory muscles in nine opossums, finding a generalized anatomical pattern with differences related to skull morphology. Variation in quantitative myological data and estimated bite force was mostly related to size, and the increase in bite force supports dietary diversification associated with size increase.

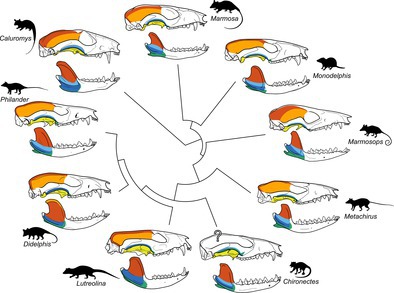

## 1 INTRODUCTION

Opossums represent the vast majority of New World marsupials, and Didelphidae (Didelphimorphia) is the largest family of marsupials, with 128 species widely distributed in the Neotropical region and occurring in a wide variety of habitats from Patagonia to southern Canada (Astúa, 2015; Astúa et al., 2023; Voss & Jansa, 2021). Some species are almost exclusively terrestrial or arboreal, while others use different forest strata equally. Additionally, the family includes the only semi‐aquatic marsupial known (Astúa, 2015; Voss & Jansa, 2021). This variation in locomotion preferences can be reflected in several features of their morphology, such as limb proportions, skeletal adaptations, or external morphology (Astúa & Guilhon, 2023; Voss & Jansa, 2021). However, didelphids are described as generalist or opportunist animals in terms of diet (Lessa et al., 2023; Vieira & Astúa de Moraes, 2003). Species show a great deal of overlap in the items consumed and can vary their diet depending on availability (Carvalho et al., 2019; Leiner & Silva, 2007; Lessa & Geise, 2014; Thielen et al., 1997). They feed on invertebrates, small vertebrates, fruits, and occasionally also on flowers and nectar, with the different levels of specialization or preference following a continuum, rather than strict categories (Astúa de Moraes et al., 2003; Lessa et al., 2023).

Morphometric analyses of the variation in size and shape in the skull of didelphids have addressed the relationship between diet and morphology of the cranium (Chemisquy et al., 2021; Astúa de Moraes et al., 2000) and mandible (Astúa de Moraes et al., 2000; Silva‐Neto et al., 2024). Despite general morphological similarities, Astúa de Moraes et al. (2000) observed differences in cranial and mandibular shape that could correspond to the main dietary trends represented in Didelphidae, towards frugivory (*Caluromys philander*), insectivory (*Metachirus nudicaudatus*), and carnivory (*Lutreolina crassicaudata*). These differences seemed to be related to variations in the masticatory musculature, as indicated by the degree of narrowing of the braincase and enlargement of the coronoid process for the temporalis muscle, and height of the angular process and development of the masseteric fossa for the masseter muscle, which may also be correlated with differences in diet. However, even though variation in cranium shape may separate species based on diet, this could be explained by other factors, such as phylogenetic affinity (Chemisquy et al., 2021).

Finding a correspondence between the anatomy of the didelphid masticatory apparatus and diet has been challenging, due either to limitations in diet quantification or to morphological inferences that focus mainly on bone structure. However, soft tissue has been shown to have functional correlations with different behaviors in other mammals, including marsupials (Dawson et al., 2014; Morales et al., 2018; Warburton, 2009; Warburton et al., 2012; Warburton et al., 2013). In mammals, muscle proportions vary according to the function of the masticatory apparatus, such as cutting, grinding, or gnawing, as well as diet (Turnbull, 1970). Changes in the orientation, internal arrangement, size, and degree of differentiation of functionally associated muscles allow a better understanding of possible adaptive evolution and selective pressures (Herring, 1993). Information on muscle architecture can also be used to estimate performance metrics, such as the capacity for muscle extension, related to muscle fiber length, and physiological cross‐sectional area (PCSA), which is proportional to muscle force (Abreu et al., 2023; Gorniak, 1985; Hartstone‐Rose et al., 2019). The trade‐off between muscle extension and force production predicts how functionally adapted the masticatory muscles are for high gape, given that they are attached to the mandible, and generating bite force, estimated as the muscle force transmitted to the bite location.

Scaling relationships of muscle mass, fiber length, PCSA, and bite force with body size have been the focus of studies in a variety of mammals (Hartstone‐Rose et al., 2012; Hartstone‐Rose et al., 2018; Hartstone‐Rose et al., 2019; Hartstone‐Rose et al., 2022). These scaling patterns lead to differences in performance (Pfaller et al., 2011) which, when unexplained by size, may be related to dietary factors such as the size and mechanical properties of the food. If body size is the main factor responsible for variation in performance, opossums are an excellent model for study, as allometry plays an important role in the morphological evolution of this group. Analyses of modularity and morphological integration have shown that marsupials have highly integrated skulls (Shirai & Marroig, 2010). This means that didelphids tend to respond to selective pressures with changes in size, as shown by the strong correlation between size and cranium shape (Chemisquy et al., 2021). However, Silva‐Neto et al. (2024) did not find a primary role for allometry in the morphological diversity of didelphid mandibles. Thus, it is unclear whether size could be the main driver of masticatory muscle variation if, just like the mandible, they have a greater relationship with masticatory performance.

Despite these questions, studies on the masticatory anatomy of opossums are scarce and almost none present quantitative data (Coues, 1872; Delupi et al., 1997; Diogo et al., 2016; Hiiemäe & Jenkins Jr., 1969; Minkoff et al., 1979; Sidebotham, 1885; Turnbull, 1970). Most analyses have concentrated on the genus *Didelphis*. Sidebotham (1885) described the muscular system of *Chironectes*, but did little to illustrate the muscles of the head, and only one comparative analysis dissected specimens from different genera, namely *Lutreolina* and *Didelphis* (Delupi et al., 1997). *Lutreolina* did not show any major differences compared to *Didelphis*, but the authors observed a greater development of the adductor muscle mass, relating these findings to the more carnivorous habits reported for *Lutreolina* compared to *Didelphis*, which is omnivorous. Apart from these descriptive studies, very little is currently known about the masticatory muscles of this highly speciose family.

This work aimed to describe the anatomy of the masticatory apparatus of a greater diversity of opossums using a comparative approach, addressing patterns of variation in muscle anatomy, muscle fiber architecture, and functional relationships for bite force performance and use of food resources. As preliminary analyses have indicated, we expect aspects of the morphology and muscular performance of the masticatory apparatus to reflect diet to some extent, as well as to be related to body size.

## 2 METHODS

### 2.1 Dissections and muscle measurements

We dissected the adductor muscles of the mandible (*m. masseter*, *m. temporalis*, *m. pterygoideus medialis*) of 14 specimens of the genera *Caluromys* (*n* = 2, *Caluromys* sp.), *Didelphis* (*n* = 3, *D. albiventris*), *Lutreolina* (*n* = 2, *L. crassicaudata*), *Philander* (*n* = 1, *P. quica*), *Metachirus* (*n* = 2, *M. myosuros*), *Marmosa* (*n* = 1, *Marmosa* sp., *n* = 1, *M. murina*), *Marmosops* (*n* = 1, *M. incanus*), and *Monodelphis* (*n* = 1, *Monodelphis* sp.) (Table 1). We also dissected an additional specimen of *Chironectes minimus* which had its head partially consumed by piranhas. All the skulls of the individuals were fresh frozen or stored in ethanol (70%) in the Mammal Collection of the Taxonomic Collections Center of the Universidade Federal de Minas Gerais and the Mammal Collection of the Universidade Federal de Pernambuco until dissection. The potential effect of ethanol storage on fresh tissue was taken into account (see below). All specimens lacked any obvious damage to the adductor musculature, except for the *Chironectes* specimen, and had a fully erupted dentition, except for three juveniles: UFMG8410, *Caluromys*; UFMG8413, *M. myosuros*; and UFMG8408, *C. minimus*. Although ontogenetic changes in the masticatory muscles and cranial sexual dimorphism have been reported for didelphids (Abreu & Astúa, 2024; Astúa, 2010), we did not make a distinction between ages or sexes due to limitations of the sample.

**TABLE 1:** Mass, average fascicle length, and pPCSA of the adductor musculature, and estimated bite forces on the first molar (m1) and canine of the specimens used in the study.

Species	ID	Age	Preservation	Cranium length (cm)	Total mass (g)	Average fascicle length (cm)	Total pPCSA (cm^2^)	Bite force at m1 (N)	Bite force at canine (N)
*Didelphis albiventris*	DAM904	Adult	Fresh/Freeze	9.976	5.76	1.080	6.215	57.508	40.164
*Didelphis albiventris*	DAM905	Adult	Fresh/Freeze	10.939	5.51	0.989	6.143	60.163	45.316
*Didelphis albiventris*	DAM903	Adult	Fresh/Freeze	11.583	12.54	1.355	10.243	117.581	89.477
*Philander quica*	UFMG2187	Adult	70% Ethanol	7.820	4.918	0.927	5.912	51.605	36.440
*Lutreolina crassicaudata*	UFMG7991	Adult	70% Ethanol	6.225	1.571	0.769	2.343	17.668	12.447
*Lutreolina crassicaudata*	UFMG7990	Adult	70% Ethanol	6.036	1.420	0.667	2.357	19.173	13.699
*Metachirus myosurus*	UFMG8413	Juvenile	70% Ethanol	5.584	0.693	0.687	1.114	8.471	5.655
*Metachirus myosurus*	UFMG2185	Adult	70% Ethanol	6.519	1.251	0.548	2.692	23.052	16.383
*Caluromys* sp.	UFMG8409	Adult	70% Ethanol	5.716	0.980	0.448	2.351	26.245	18.310
*Caluromys* sp.	UFMG8410	Juvenile	70% Ethanol	5.238	1.082	0.519	2.249	20.316	13.952
*Marmosa* sp.	UFMG8411	Adult	70% Ethanol	4.934	1.927	0.660	3.170	21.401	14.996
*Marmosa murina*	UFMG8412	Adult	70% Ethanol	3.624	0.275	0.388	0.804	5.755	4.126
*Marmosops incanus*	UFMG8406	Adult	70% Ethanol	4.471	0.642	0.471	1.521	11.761	0.449
*Monodelphis* sp.	UFMG8403	Adult	70% Ethanol	4.032	0.422	0.506	0.943	7.617	0.261
*Chironectes minimus*	UFMG8408	Juvenile	70% Ethanol	7.461					

We removed the muscles under a binocular stereomicroscope and with the mandible occluded or nearly occluded (<5° between the temporo‐mandibular articulation and the upper and lower incisors). We observed and recorded the origin and insertion areas, along with fiber orientations. The *m. pterygoideus lateralis* was kept intact during dissections for better stabilization of the mandible. As it may act opening instead of closing the mandible (Herrel et al., 2024) and it contributes very little to the masticatory muscle mass (Turnbull, 1970), it is of lesser importance for our main goals here. Immediately after removal, we transferred the muscles to 70% ethanol to avoid dehydration prior to weighing (Martin et al., 2020). They were then left to dry excess liquid on absorbent paper and weighed on scales of 0.01 g (Marte AD3300) or 0.0001 g (Shimadzu AY220) precision. We submerged each muscle in a water solution of sulfuric acid (10%), and they were heated at 60°C until the connective tissue between fascicles was dissolved (usually 15–60 min). Muscles were left to dry slightly on absorbent paper and we transferred them to a water glycerin solution (50%), with due caution to avoid damage to the fibers. We randomly selected 5–10 fascicles (representing fiber lengths), photographed them, and measured them using ImageJ 1.53 k (Schneider et al., 2012). We then used the average length of the measured fascicles for each muscle for the PCSA calculations. We adjusted the muscle masses for those specimens stored in ethanol using a correction factor of 1.69, following Leonard et al. (2022a) (but no formalin pre‐fixation was used in our sample). The fascicle length does not significantly change while muscles are still attached to the bone (Leonard et al., 2022b), thus we did not use any correction for those. We calculated the potential PCSA (pPCSA) by dividing the volume (*v*) with the mass values (*m*) and a density (*ρ*) of 1.06 g cm^−3^ (Leonard et al., 2020; Mendez & Keys, 1960)—by the average fascicle length (*fl*),

$$\text{pPCSA}=\frac{v}{\mathrm{fl}}\Leftrightarrow \text{pPCSA}=\frac{m}{\rho \times \mathrm{fl}}.$$

We have labeled our estimates as pPCSA because we have not corrected it for pennation angle, to remove the force component transverse to the line of action of the muscles (Martin et al., 2020). Thus, our results are overestimations of the actual PCSA (they would be the actual PCSA if there was no pennation), and are labeled here differently for clarity. Although pennation angle adjusts the PCSA for the arrangement of packing of the fascicles and has strong implications for muscle performance (Holt et al., 2016), we did not take it into account due to the practical difficulties in measuring the angle in masticatory muscles, as addressed by Hartstone‐Rose et al. (2018) and Laird et al. (2020). We only measured the muscles on one side of the head, and we only used the opposite side when there was some damage during the dissection or due to the excessive action of the acid solution.

### 2.2 Bite force estimation

We used a two‐dimensional lever model to estimate bite force. The muscles act along a rigid bar (the mandible) between the articulation and the teeth, where resistance is met (e.g., food) (Greaves, 2012). The moment of a lever is the product of the force and the perpendicular distance between that force and the axis of rotation. This distance also is called the moment arm or lever arm. The muscle force (acting force) and the reaction force at the biting point (or food reaction force) generate opposing moments for the mandible rotation (Herrel et al., 1998a; Herrel et al., 1998b; Herrel et al., 2008). The vector of the bite force is equal to that of the food reaction force in magnitude and opposite in sense (Brassard, Merlin, Guintard, et al., 2020). We calculated the bite force (${\vec{F}}_{\text{bite}}$) based on the sum of the moments of each muscle ($\sum {\vec{M}}_{\text{muscles}}$) and the moment of the bite force (${\vec{M}}_{\text{bite}}$) unilaterally, which are equal with the mandible in equilibrium,

$${\vec{M}}_{\text{bite}}=\sum {\vec{M}}_{\text{muscles}},$$

$$\Leftrightarrow F_{\text{bite}}\times d_{\text{bite}}=\sum {\vec{M}}_{\text{muscles}},$$

$$\Leftrightarrow F_{\text{bite}}=\frac{\sum {\vec{M}}_{\text{muscles}}}{d_{\text{bite}}}.$$

The moment of the muscle force of each muscle was determined by the pPCSA multiplied by a constant muscular tension of 31 N/cm^2^ (${\vec{F}}_{\text{muscle}}$), close to the average described for *Didelphis virginiana* (Thomason et al., 1990), with its respective moment arm or in lever ($d_{\text{muscle}}$) calculated as the perpendicular distance from the temporo‐mandibular articulation to the vector of the line of muscle action between the centroids of the attachment areas of the muscle on the cranium and the mandible. The moment arm of the bite force or out‐lever ($d_{\text{bite}}$) was estimated perpendicular to the vector of the food reaction force at the bite point (either the canine or the first molar, in our case). We intersected the force vector at an angle of 90° relative to the axis connecting the temporo‐mandibular articulation and the tip of the anteriormost lower incisors. The muscle attachment centroids and moment arms were estimated using ImageJ 1.53 k (Schneider et al., 2012) from lateral photographs of the articulated cranium and mandible in the same position as the skulls were dissected. We used the observations of the specimens and conspecifics to estimate the area of origin of the *m. pterygoideus medialis* in the lateral view.

We calculated the bite force at the canine and at the first molar separately. We did not consider a bilateral biting because Didelphidae bite unilaterally and have an unfused mandibular symphysis, allowing for independent movements of the hemi‐mandibles during mastication (Bhullar et al., 2019), making it uncertain to assess how much force is being transmitted from the muscles of the balancing side, but a stronger influence of the muscles on the working side is expected (Crompton, 1981; Davis et al., 2024).

### 2.3 Analyses

We analyzed the allometric relationships of all the variables using reduced major axis (RMA) regressions, using cranium length as the size proxy. We measured cranium length as the linear distance between the anterior end of the rostrum and the posterior end of the interparietal, on the sagittal line, from dorsal photographs of the cranium, as a proxy for skull size. Although body mass was not available for most specimens, skull size is consistently correlated with body size in mammals (Emerson & Bramble, 1993; Mitchell et al., 2024), and similar allometric patterns between the two measurements were also recorded for *Didelphis albiventris* (Abreu & Astúa, 2024) and across opossums (Pilatti & Astúa, 2017). Mass, pPCSA, and bite forces scale differently from cranium length and were thus linearized (i.e., using their cube or square root) to standardize the isometric slope equal to 1 (Abreu et al., 2023; Deeming et al., 2022; Hartstone‐Rose et al., 2022). All data were log‐transformed before analyses. We conducted regressions of cranium length with the mass, average fascicle length, and pPCSA of the adductor groups (*m. masseter*, *m. temporalis*, *m. pterygoideus medialis*) and of the total adductor musculature, and the bite forces on the first molar and canine. We obtained the mass and pPCSA of the adductor groups with more than one muscle layer and of the entire adductor musculature by adding up the values of the individual muscles. Average fascicle length of the adductor groups with more than one muscle layer and of the entire adductor musculature was calculated using a weighted average with muscle mass (Hartstone‐Rose et al., 2022). We also performed bite force regressions with the mass, average fascicle length, pPCSA, and moment of force of the muscle groups to determine the main drivers of bite force. The coefficient of determination (*R* ^2^) was used as a criterion for selecting the models that explain the variation in bite force. Only the bite force on the first molar was used in these regressions due to the functional relevance of the bite site (Santana et al., 2022; Thomason et al., 1990) and to avoid reducing statistical power.

**FIGURE 1:**
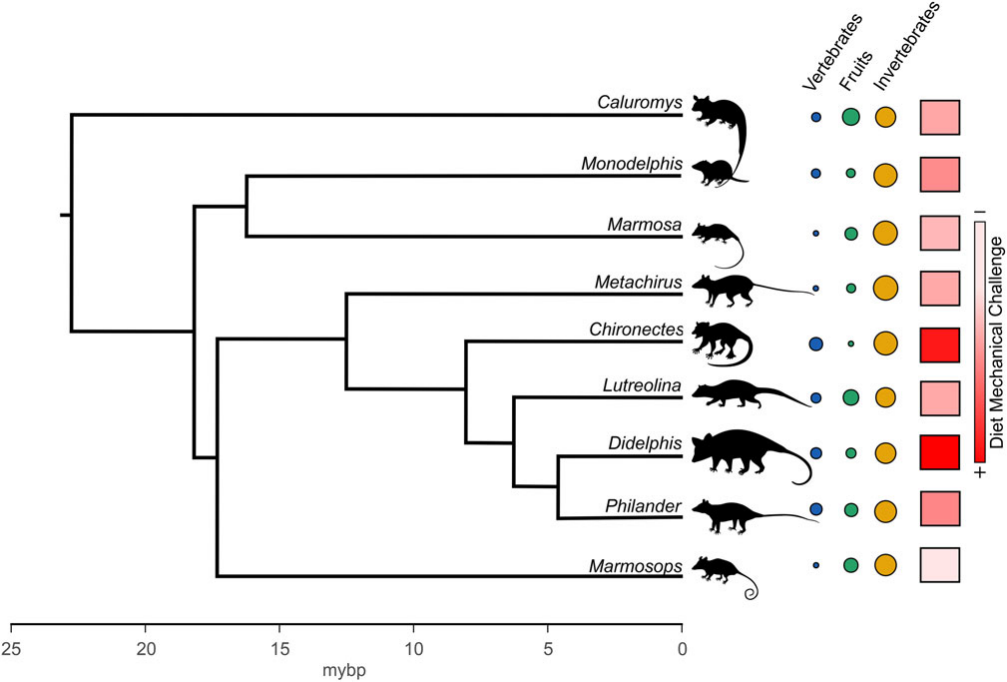
Genera included in this study, with their phylogenetic relationships and diet variables estimates. Phylogeny from Silva‐Neto et al. (2024) pruned to the genera included here, scaled to time. Items in the diet are scaled to importance and diet mechanical challenge for each genus (see text for further details). mybp: million years before present. Genera silhouettes by M. Cavalcanti, P. Pilatti, and D. Astúa, available on www.phylopic.org (not to scale).

To test the relationship between bite force and diet, we organized a diet composition matrix with the genera. We assigned the food items into four categories: invertebrates, vertebrates, fruits, and plants (leaves and flowers). The frequency of items consumed was reviewed from literature studies on each genus (Andrade, 2017; Busch & Kravetz, 1991; Caceres, 2004; Cáceres, 2002; Carvalho et al., 2005; Casella & Cáceres, 2006; Castilheiro & Santos Filho, 2013; Facure & Ramos, 2011; Kuhnen et al., 2017; Leiner & Silva, 2007; Lessa & Costa, 2010; Lessa & Geise, 2014; Macedo et al., 2010; Parreira Claro & Hannibal, 2022; Santori et al., 1997). Non‐natural items identified as garbage or human food were not counted. In those cases where there was a distinction in the frequency of items between seasons and the general pattern was provided, only the latter was used. The relative frequency of each item was calculated from the weighted average of the frequencies of occurrence and the sample size of the studies. The 10 relatively most frequent items in the diet of each genus have been separated. Invertebrates made up the majority of the diet in all genera (Figure 1). The consumption of vertebrates and fruits was more variable, but we avoided assigning discrete dietary categories (e.g., insectivores‐frugivores, insectivores‐carnivores) as all genera show some degree of overlap, as already noted by Vieira and Astúa de Moraes (2003) and Lessa et al. (2023). We then conducted a principal coordinates analysis with the *pcoa* function from the *ape* package (Paradis & Schliep, 2019), using the Euclidean distances between genera and the relative frequency of each category arcsine‐transformed (Grossnickle, 2020a; Navalon et al., 2019). We used the vectors of the first axis in a Spearman correlation analysis with the bite force of each genus. As bite force is influenced by body size, we corrected the estimates for cranium length using the residuals of the regression with bite force (on the first molar and canine) and cranium length, free of the variation associated with allometry. We used the average of the two variables when a genus was represented by more than one specimen.

In addition to diet composition, we also used information on the material properties of food items to enhance our understanding of the exploitation of these resources, especially the diet mechanical challenge (the higher the proportion of hard items, the harder the challenge) that individuals must deal with when consuming certain items. Sibbing (1991), based on Lucas and Luke (1984), delineated four main categories of foods based on their mechanical properties and appropriate processing mechanisms: hard brittle, turgid, soft tough, and tough fibrous. Two properties considered are possibly relevant to biting: strength, which indicates the stress the food can withstand before deformation and the onset of breakage, and toughness, a measure of the food's resistance to breakage growth (Berthaume, 2016; Lucas, 2004). Items in the hard brittle and soft tough categories (hereafter referred to as hard and soft) stand out with high strength and low toughness, and moderate strength and high toughness, respectively. We considered the relevance of these hard and soft items as indicative of the mechanical demands of the diet. To do this, we take into account the 10 most frequent items in the first matrix and their relative frequency. The items were distributed into four categories: hard brittle (bones, some adult insects, and dried fruit), turgid (insect larvae), soft tough (some insects and fleshy fruit) and tough fibrous (leaves and flowers). Additional information for food classification was taken from Aguirre et al. (2003), Dumont (2006), Cakenberghe et al. (2002), and Verwaijen et al. (2002). Unidentified arthropods and fruits were not counted. Finally, the frequency of hard items relative to the frequency of soft items for each genus was used to represent the diet mechanical challenge continuously. We conducted a Spearman correlation analysis with these log‐transformed values and the bite force (on the first molar and canine) corrected for size, using the method described previously.

All regressions were conducted in PAST 4.03 (Hammer et al., 2001), but the remaining analyses were carried out in R (R Core Team, 2021). We considered the significance level to be 0.001 due to a series of consecutive tests, and moderate evidence for p between 0.001–0.05. Deviations from isometry were indicated by the 99.9% confidence interval. We also considered moderate evidence for deviations at 95% confidence intervals.

## 3 RESULTS

### 3.1 Anatomical description

**FIGURE 2:**
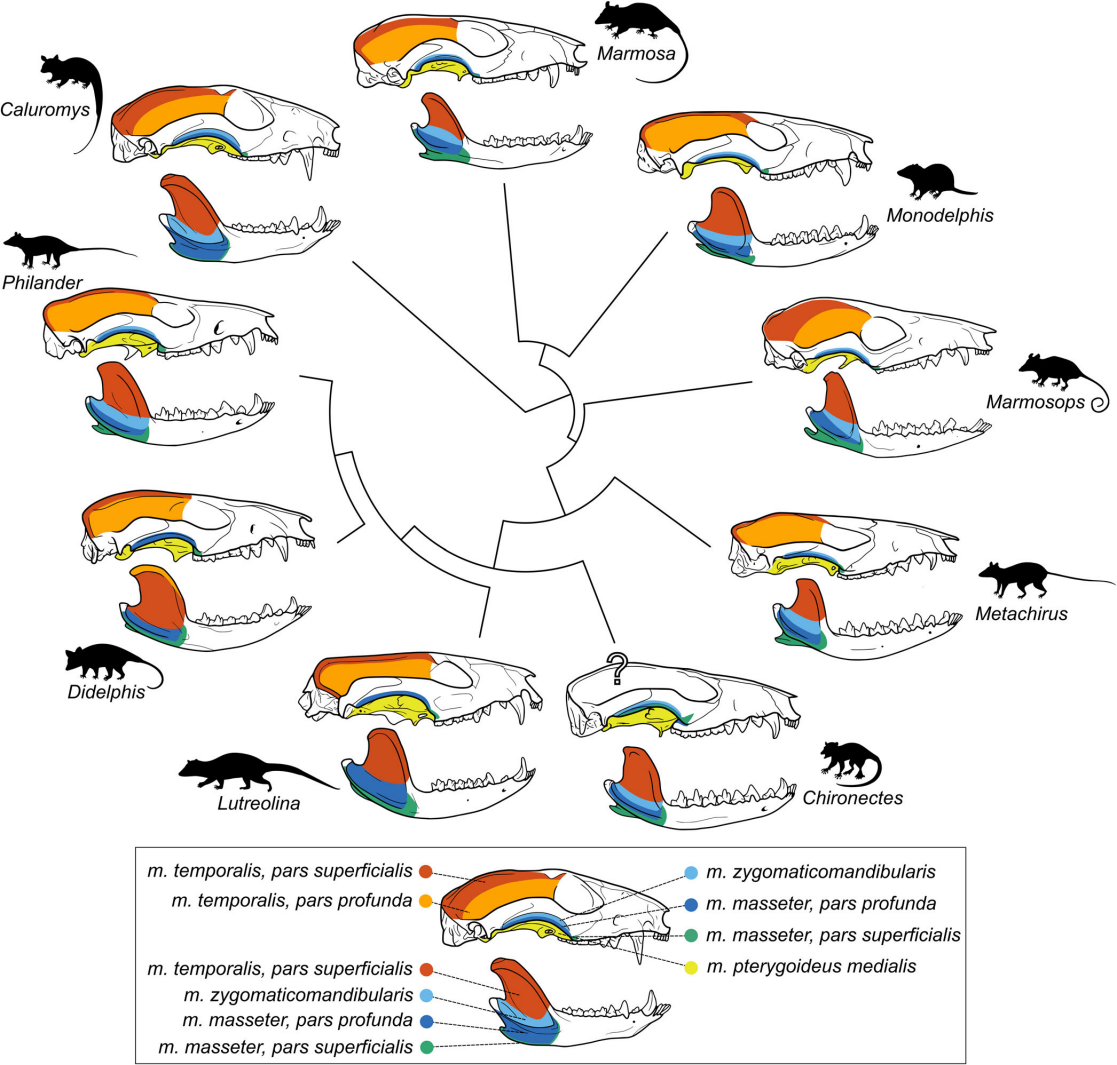
Attachment areas of the adductor muscles in the cranium and mandible, in lateral view, for the indicated Didelphidae genera. Information on part of the muscles for *C. minimus* is missing, as the *temporalis* of the dissected specimen was damaged. Phylogeny from Silva‐Neto et al. (2024) pruned to the genera included here. Genera silhouettes by M. Cavalcanti, P. Pilatti, and D. Astúa, available on www.phylopic.org.

The lateral view of the anatomy and attachment areas of the muscles is mapped in Figure 2. Photographs of dissection in *Didelphis*, *Philander*, and *Caluromys* are available in Figures [Link], [Link], Supporting Information. When a description does not mention any specific genus, it applies to all genera included in this analysis. The same description also applies at the genus level when the specimen is not identified.

#### 3.1.1 Masseter (*m. masseter*)

**FIGURE 3:**
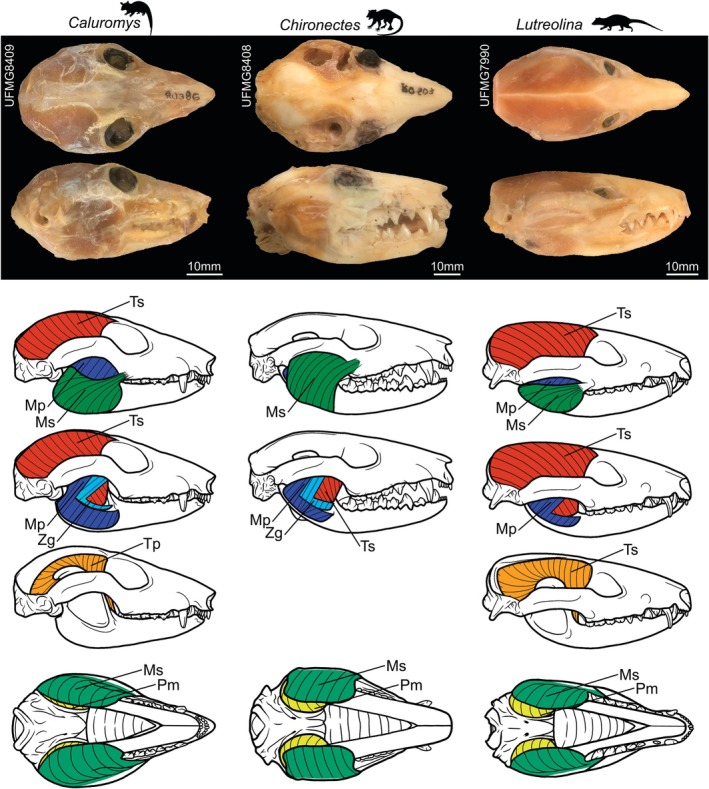
Photographs of superficial adductor musculature in *Caluromys*, *Chironectes*, and *Lutreolina* in dorsal and lateral views, and adductor muscles illustrated in lateral and ventral views. Ts, *temporalis pars superficialis*; Tp, *temporalis pars profunda*; Ms, *masseter pars superficialis*; Mp, *masseter pars profunda*; Zg, *zygomaticomandibularis*; Pm, *pterygoideus medialis*.

**FIGURE 4:**
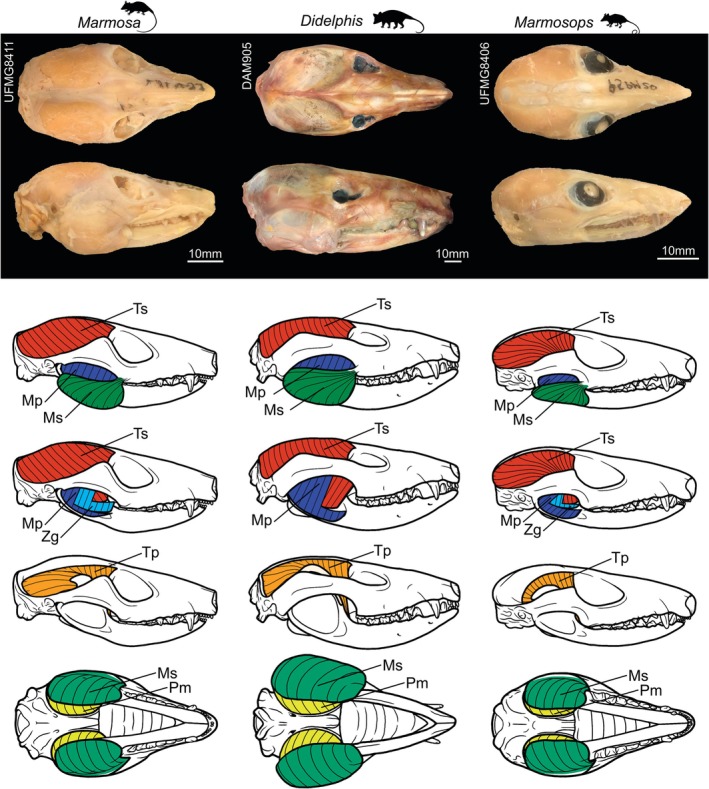
Photographs of superficial adductor musculature in *Marmosa*, *Didelphis*, and *Marmosops* in dorsal and lateral views, and adductor muscles illustrated in lateral and ventral views. Ts, *temporalis pars superficialis*; Tp, *temporalis pars profunda*; Ms, *masseter pars superficialis*; Mp, *masseter pars profunda*; Zg, *zygomaticomandibularis*; Pm, *pterygoideus medialis*.

**FIGURE 5:**
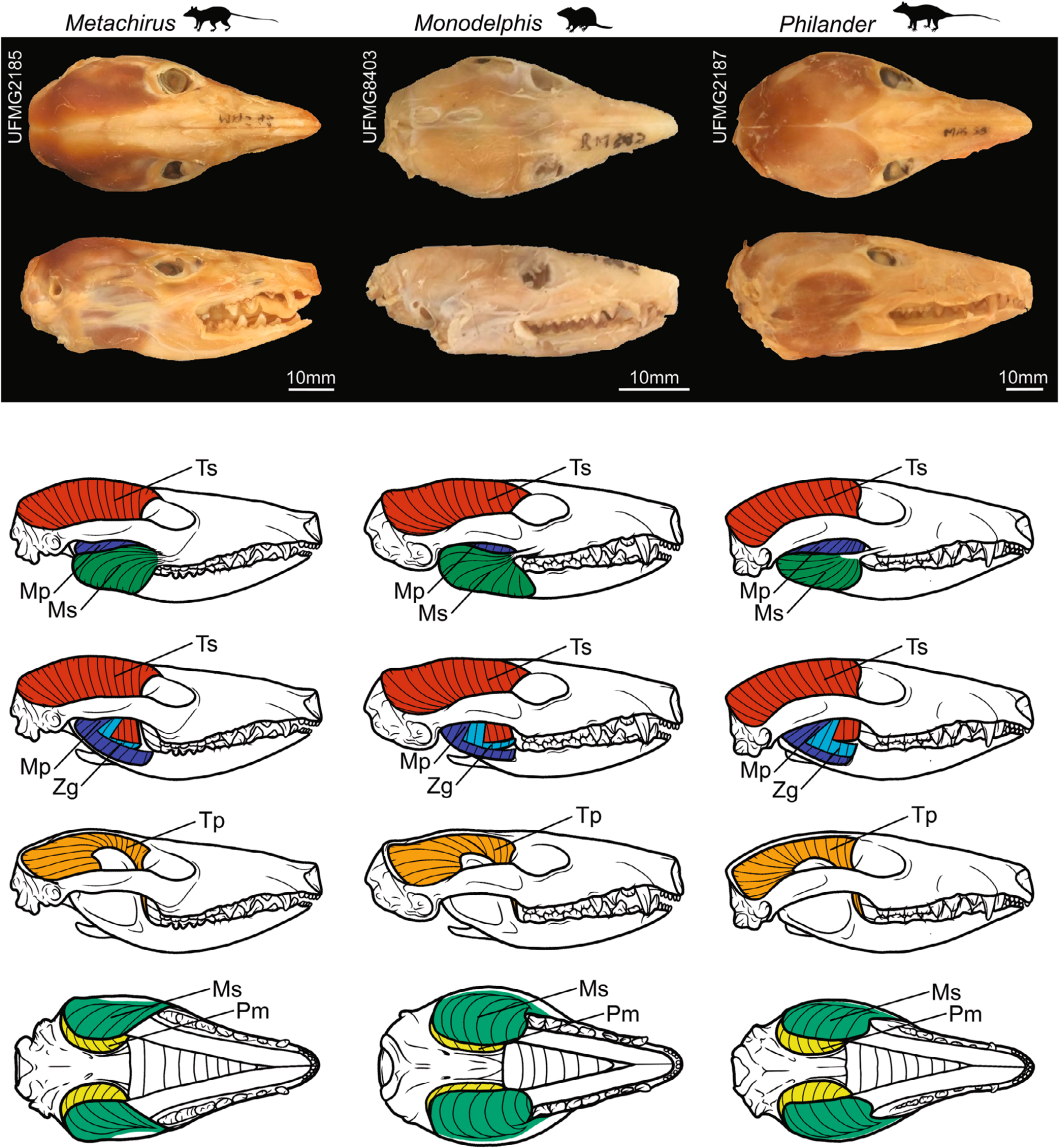
Photographs of superficial adductor musculature in *Metachirus*, *Monodelphis*, and *Philander* in dorsal and lateral views, and adductor muscles illustrated in lateral and ventral views. Ts, *temporalis pars superficialis*; Tp, *temporalis pars profunda*; Ms, *masseter pars superficialis*; Mp, *masseter pars profunda*; Zg, *zygomaticomandibularis*; Pm, *pterygoideus medialis*.

In all genera the masseter had two layers, one superficial (*m. masseter pars superficialis*) and one deep (*m. masseter pars profunda*). The superficial masseter originated posteriorly on the maxilla, just ventral to the jugal and dorsal to the last upper molars (M3‐M4) in almost all specimens, and inserted onto the angular process of the mandible (Figures 2, 3, 4, 5). The origin was on a bony prominence and was marked by a strong tendon in *Didelphis*, *Philander*, *Lutreolina*, and *Caluromys*. Some fibers extended slightly more posteriorly over the jugal on the zygomatic arch in *Lutreolina* and *Metachirus*, and more anteriorly on the maxilla, near or over the *m. levator labii superioris* in *Didelphis*, *Philander*, *Lutreolina*, and *Caluromys*. The superficial masseter in *Chironectes* originated particularly along the jugal and ventralmost part of the lateral face of the zygomatic arch (Figure 2). The fibers in this specimen were more vertical when they inserted posteroventrally from their origin on the cranium onto the angular process (Figure 3). In other genera, the more posterior the insertion of the fibers, the more horizontal the orientation of the fibers (Figures 3, 4, 5). This feature was less marked in *Caluromys*. In this genus, the belly of the superficial masseter was positioned more ventrally, similar to *Marmosa* (UFMG8411), but the whole muscle represented a uniform muscle layer along its length, while there was a greater accumulation of fibers near the caudal edge of the mandible and inflection of the angular process in the other genera (the angular process is not inflected in *Caluromys*). The area of insertion of the superficial masseter was also much reduced in *Caluromys* and was restricted to the ventral edge of the angular process (Figure 2). The fibers did not reach the masseteric line formed by the posterior support of the masseteric fossa, which consistently represented the boundary of the superficial and deep layers of the masseter in other genera (Figure 2).

The deep masseter originated on the ventral portion of the medial face of the zygomatic arch and inserted onto the masseteric fossa of the mandible in all the specimens, possibly involving the edge of the masseteric line, except in *Caluromys*, in which it inserted more ventrally, close to the edge of the angular process (Figures 2, 3, 4, 5). The origin extends from the region between the maxilla and the jugal to just anterior to the temporomandibular joint (Figure 2). The most anterior fibers may start from the ventral margin of the anterior end of the zygomatic arch, as in *Didelphis* and *Marmosa* (UFMG8411), and more markedly in *Chironectes* (Figure 2). This muscle was particularly bulging in this *Marmosa* specimen. The fibers of the deep masseter have a more vertical path than the superficial masseter but are still oriented anteriorly. In *Metachirus*, there was a marked change in fiber orientation: the fibers of the most anterior third of the muscle were approximately vertical.

A zygomasseteric complex was observed when an additional muscle layer was distinguished from the deep portion of the masseter, present in most specimens, identified as zygomaticomandibular (*m. zygomaticomandibularis*). It originated above the deep masseter on the medial surface of the zygomatic arch and inserted deeper onto the masseteric fossa, except in *Caluromys*, in which it filled the entire masseteric fossa (Figures 2, 3, 4, 5). The muscle generally represented a layer thinner than the deep masseter, except in *Chironectes*, in which it was more voluminous than the deep masseter. The deeper fibers of the zygomaticomandibular showed some degree of continuity with the fibers of the superficial temporalis. Only *Caluromys* (UFMG8410) showed a full separation of the zygomaticomandibular, which inserted partially through an aponeurosis on the caudal portion of the masseteric fossa. The path of the zygomaticomandibular fibers was usually slightly more vertical than that of the deep masseter, but they acquired a more anterior orientation in *Caluromys*. Only *Didelphis* and *Lutreolina* did not show any clear distinction of the zygomaticomandibular. We did find fibers slightly more vertical than the deep masseter that blended with the insertion of the superficial temporalis in *Didelphis*, but the removal of the deep masseter in *Lutreolina* already results in posteriorly oriented fibers that are characteristic of the temporalis. The insertion of the deep masseter in these two genera was more extensive over the masseteric fossa (Figure 2).

#### 3.1.2 Temporalis (*m. temporalis*)

The temporalis, like the masseter, has two distinct portions, a superficial (*m. temporalis pars superficialis*) and a deep one (*m. temporalis pars profunda*), in all genera. The superficial temporalis originates from the temporal fascia and inserts on the lateral surface of the coronoid process (Figures 2, 3, 4, 5). The fibers run anteroventrally (i.e., are oriented posteriorly), but deviate their path at the level of the zygomatic arch, resulting in a slightly more anterior orientation in the insertion in *Didelphis*, or less posterior in the other genera. The path of the fibers was also less posteriorly directed as the fibers approached the orbit (Figures 3, 4, 5). The origin of the fibers extended dorsally on the cranium to attach to the sagittal crest in *Didelphis*, *Lutreolina*, *Philander*, and *Monodelphis* (Figures 3, 4, 5). *Chironectes* also has a sagittal crest, but the temporalis did not appear to attach to it. However, it is difficult to reliably describe the extent of the temporalis in this genus as the specimen available was partially predated and thus damaged. *Marmosa*, *Caluromys*, *Marmosops*, and *Metachirus* lack a sagittal crest. The superficial temporalis did not extend to the midline of the cranium in these specimens. The upper limit corresponds to the temporal line, which curves towards the midline but does not converge between sides as the sagittal crest (Figures 3, 4, 5). The temporal line, together with the post‐orbital process, delimited the superficial temporalis anteriorly, which is also evident in *Chironectes*. The more caudal fibers originated on the nuchal crest and then tended to be oriented more posteriorly. The sagittal crest in *Lutreolina*, which remains at the same height throughout its length, results in a relatively more dorsal origin of the posterior fibers. The insertion areas covered the coronoid process to its base between the condyle and the notch of the horizontal ramus of the mandible (Figure 2). The insertion extended further inferiorly on the mandibular ramus in *Didelphis* (Figure 2). However, the greatest accumulation of fibers always occurred at the top of the coronoid process, between the process and the dorsalmost region of the zygomatic arch, in all genera.

The deep temporalis originates posterior to the orbit, in the post‐orbital constriction and temporal fossa, and inserts on the medial surface of the coronoid process (Figures 2, 3, 4, 5), dorsal to the insertion of the *m. pterygoideus medialis*. The insertion could reach the posterior edge of the top of the coronoid process, but not as in *Didelphis*, where the attachment extended to the lateral edge of the coronoid process by an aponeurosis (Figures 2 and 4). It was unclear whether this feature was exclusive to *Didelphis* or whether it was less evident in the other genera due to preservation in alcohol since *Didelphis* was the only genus dissected fresh. The insertion generally extended medially on the coronoid process to near the level of the tooth row, but was more ventral in *Caluromys*. Like the superficial temporalis, the deep temporalis also extended along the lateral wall of the cranium towards the sagittal crest, or more towards the top of the cranium when the sagittal crest was absent, and the nuchal crest. It reached the sagittal crest in *Didelphis* but not in *Lutreolina*, *Philander*, or *Monodelphis*, although it comes very close in the latter two. The more dorsocaudal extension of the deep temporalis restricted the area of bony attachment of the superficial temporalis in *Didelphis*, *Philander*, and *Monodelphis*, over the sagittal and nuchal crests (Figure 2). The fibers tended to be concentrated in the post‐orbital constriction, especially in genera with pronounced posterior cranium flexion (*Caluromys* and *Marmosops*) (Figures 2, 3, 4). Fibers located more caudally, especially dorsal to the external acoustic meatus, were prominent (except in *Marmosops*) and had more posterior orientations, while they acquired a more pronounced vertical path as they approached the orbit (Figures 3, 4, 5). The change in fiber orientation was very marked in *Monodelphis* and *Marmosa*, distinguishing an anterior portion with a more vertical fiber path and a posterior portion, with the fibers oriented more posteriorly.

#### 3.1.3 Medial pterygoid (*m. pterygoideus medialis*)

The medial pterygoid originates on the pterygoid bone, the alisphenoid bone, and the posterolateral region of the palate (Figures 3, 4, 5). The fibers run posteroventrally (i.e., are oriented anteriorly) to insert onto the angular process on the medial side of the mandible. The attachment sites did not vary across genera, but the areas were influenced by the arrangement of the pterygoid and palatine surfaces at the base of the cranium and the morphology of the angular process. The lateral surface of the pterygoid and part of the palatine where the medial pterygoid arises are positioned more laterally in relation to the presphenoid and basisphenoid in *Caluromys* and *Monodelphis*, which reduces the area exposed for the muscle to attach onto the cranium (Figures 3 and 5). *Caluromys* also differs in lacking an inflexed angular process. The insertion of the medial pterygoid in this genus was more inferior in relation to the horizontal ramus of the mandible. The fibers traveled a relatively longer path that deviated little mediolaterally compared to the other genera, in which the fibers tended to run more laterally from the origin to insert along the support formed by the inflexed angular process and the vertical ramus of the mandible, ventral to the level of the temporomandibular joint. The medial pterygoid in these other genera was relatively more voluminous due to the additional fibers that are inserted along the medial extension of the process.

### 3.2 Proportion of masticatory muscles

**FIGURE 6:**
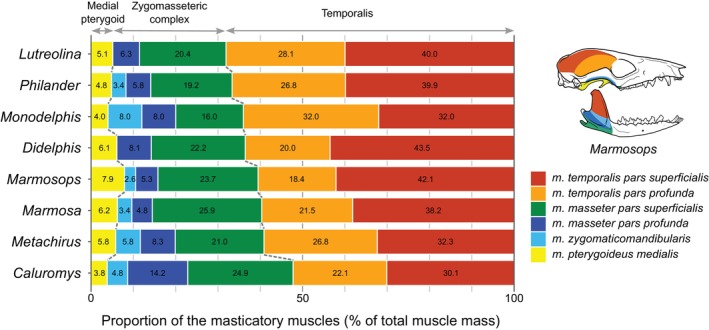
Relative proportions of the adductor muscles (with values indicated directly on the bars) in Didelphidae genera examined.

The overall masses of the adductor muscles are presented in Table 1 (the individual masses of these muscles are available in Table S1). The average proportion of muscles for each genus is shown in Figure 6. The distribution of masses indicates a greater dominance of the temporalis, followed by the zygomasseteric complex (hereafter represented by the masseter plus the zygomaticomandibular, or just the masseter when the latter was not distinguished) and the medial pterygoid. The temporalis represented more than half of the adductor musculature in all genera (52.2%–68.1%). The zygomasseteric complex varied between 26.8% and 43.9%, while the medial pterygoid was more conserved (3.8%–7.9%). *Caluromys* and *Lutreolina* were located at the opposed ends on the gradient of relative importance of the temporalis versus the zygomasseteric complex in the adductor musculature: 52.2% and 43.9% in *Caluromys*, and 68.1% and 26.8% in *Lutreolina*, respectively. *Philander* and *Didelphis* also presented high temporalis ratios (66.7% and 63.5%, respectively). The superficial portions contributed a greater fraction of the adductor mass of the muscle groups (temporalis and the zygomasseteric complex), except for *Monodelphis*, which presented a similar proportion of the two layers of the temporalis (32%). The medial pterygoid was relatively smaller in *Caluromys* and *Monodelphis* (3.8% and 4%, respectively), and larger in *Marmosops* (7.9).

### 3.3 Scaling of muscle properties and bite forces

**FIGURE 7:**
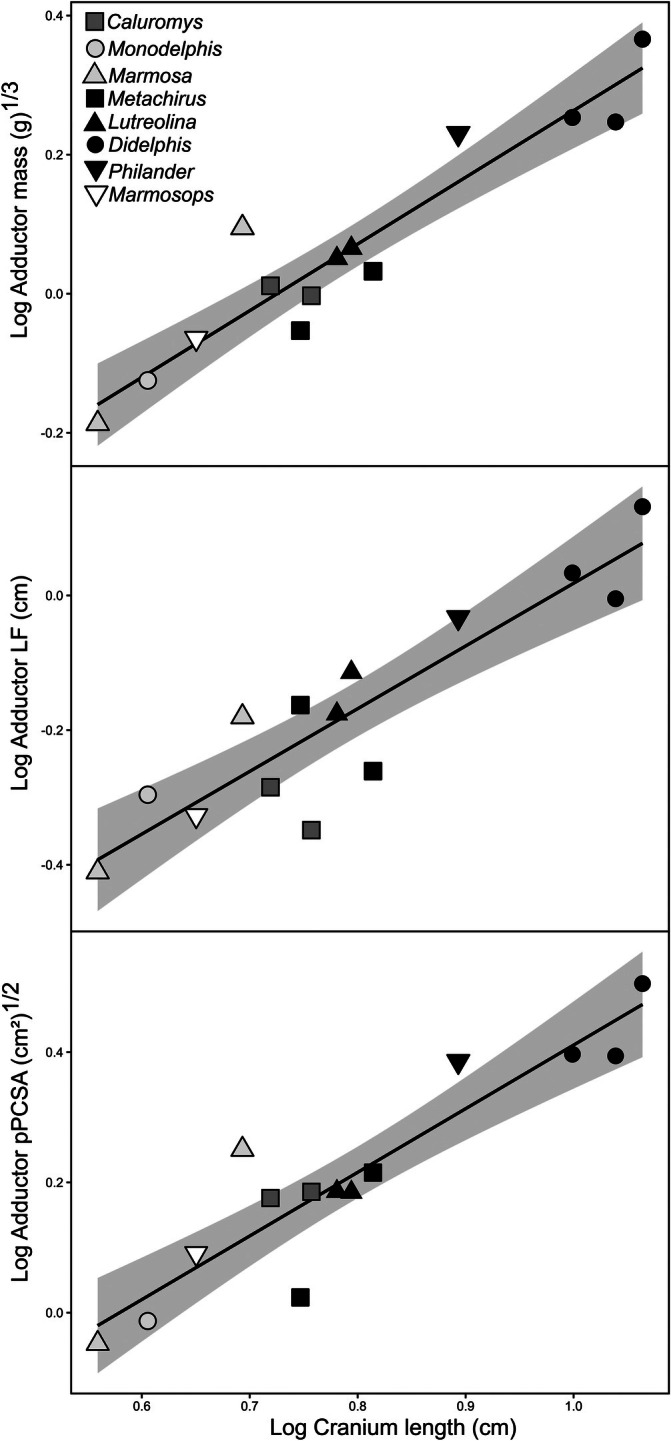
Regression of the mass, average length of the fascicles, and pPCSA of the adductor musculature onto cranium length.

**FIGURE 8:**
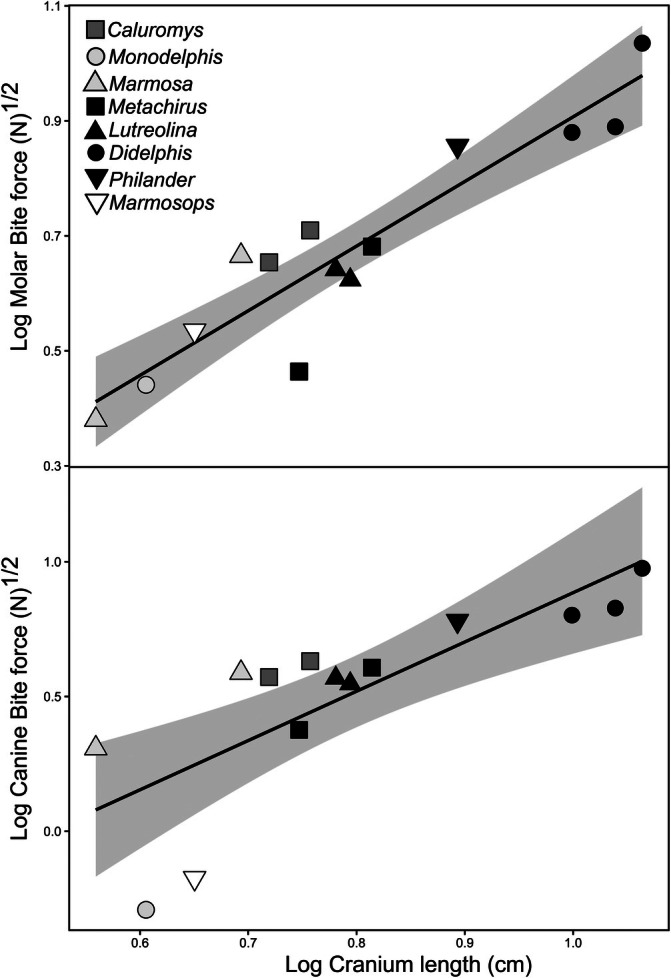
Regression of the bite forces estimated at the first molar and at the canine onto cranium length.

The regression statistics are shown in Table 2. Cranium length was a good predictor of muscle mass (*R* ^2^ = >0.8, *p* < 0.001), pPCSA (*R* ^2^ = >0.7, *p* < 0.001), mean length of temporalis fascicles, zygomasseteric complex, and adductor musculature (*R* ^2^ = >0.7, *p* < 0.001), bite force on the first molar (*R* ^2^ = 0.863, *p* < 0.001), and, more moderately, for the length of the fascicles of the medial pterygoid (*R* ^2^ = 0.688, *p* < 0.001) and bite force on the canine (*R* ^2^ = 0.627, *p* < 0.001) (Figures 7 and 8). The slopes were not different from those expected for isometry in all variables (99.9% confidence interval, CI). Only the bite force on the canine showed moderate evidence for positive allometry (95% CI = 1.422–3.197). The pPCSA of the zygomasseteric complex was the best predictor of bite force on the first molar (*R* ^2^ = 0.968, *p* < 0.001). The pPCSA of the temporalis, the mass of the zygomasseteric complex, and the moment exerted by the zygomasseteric complex and the temporalis were still highly explanatory (*R* ^2^ = >0.94, *p* < 0.001), followed by the mass of the temporalis (*R* ^2^ = 0.917, *p* < 0.001).

**TABLE 2:** Statistics of the RMA regressions with the mass, average fascicle length (FL), pPCSA, bite force at the first molar (m1) and canine onto cranium length.

Variable	Intercept	*b*	*r*	*R* ^2^	99.9% CI	95% CI
*Temporalis* mass	−0.843	1.057	0.934	0.873	0.587–1.527	0.820–1.294
Zygomasseteric mass	−0.864	0.961	0.940	0.884	0.577–1.344	0.764–1.157
*Pterygoideus medialis* mass	−1.175	1.029	0.945	0.893	0.635–1.424	0.827–1.231
Adductor mass	−0.741	1.017	0.943	0.890	0.596–1.437	0.805–1.229
*Temporalis* FL	−0.906	0.948	0.894	0.798	0.418–1.479	0.681–1.216
Zygomasseteric FL	−1.131	1.199	0.917	0.841	0.639–1.758	0.912–1.485
*Pterygoideus medialis* FL	−1.195	0.909	0.830	0.688	0.315–1.503	0.605–1.213
Adductor FL	−0.989	1.027	0.906	0.821	0.486–1.568	0.754–1.300
*Temporalis* pPCSA	−0.829	1.154	0.915	0.838	0.574–1.733	0.861–1.446
Zygomasseteric pPCSA	−0.774	0.919	0.873	0.761	0.393–1.444	0.650–1.187
*Pterygoideus* pPCSA	−1.288	1.260	0.859	0.737	0.504–2.016	0.873–1.647
Adductor pPCSA	−0.634	1.062	0.920	0.846	0.543–1.582	0.800–1.324
Bite force at m1	−0.285	1.209	0.929	0.863	0.652–1.766	0.928–1.491
Bite force at canine	−1.325	2.309	0.792	0.627	0.551–4.067	1.422–3.197

*Note*: All regressions were significant (*p* < 0.001).

Abbreviation: CI, confidence interval.

### 3.4 Bite force and diet

There was no evidence of correlation of bite force residual with diet (on the first molar, *ρ* = −0.357, *ρ* = 0.389; on the canine, *ρ* = −0.19, *p* = 0.665) or diet mechanical challenge (on the first molar, *ρ* = −0.071, *p* = 0.882; on the canine, *ρ* = −0.381, *p* = 0.36).

## 4 DISCUSSION

In this study, we provide for the first time a comparative description and quantitative information on jaw adductor muscles in nine genera of Didelphidae, five of which had never been previously described. In all of them, we identified the temporalis (superficial and deep layers), the masseter (superficial and deep layers), and the medial pterygoid muscles. The zygomaticomandibular muscle was identified in several genera, but not all, indicating variation across taxa and highlighting increased muscle complexity in the family. The temporalis was the dominant muscle and showed an inverse relation with the zygomasseteric complex, which was evident comparing *Lutreolina* and *Caluromys*, genera located at the extremes of the Didelphidae feeding gradient (Lessa et al., 2023; Vieira & Astúa de Moraes, 2003). As muscle architecture is key to understanding muscle function (Kikuchi, 2010), we obtained the length of the fascicles and the pPCSA of all muscles, and the latter was used to calculate the bite force on the canine and first molar to assess functional adaptations to trophic activity. The size, the average length of the fascicles, and the pPCSA of the muscle groups (temporalis, zygomasseteric complex, and medial pterygoid) and the entire adductor musculature scaled isometrically with size. The variation in bite forces was also a function of body size and was uncorrelated with diet and diet mechanical challenge across genera.

### 4.1 Opossum masticatory muscles anatomy

The masticatory apparatus of opossums has been classified as generalized when compared to other mammals, since the pioneering work of Turnbull (1970). More recently, Ercoli et al. (2023) constructed a new scheme of masticatory morphotypes with a specific category for didelphimorphs, characterized by a highly developed temporalis, a simplified and reduced masseter, and a reduced pterygoid. However, *Didelphis* was the only genus represented in their analyses, as it remained until now the only genus with detailed and quantitative descriptions of the masticatory muscles (Diogo et al., 2016; Hiiemäe & Jenkins Jr., 1969; Turnbull, 1970). With the dissection of new genera, the diversity of the masticatory myology of opossums can be better understood. The average proportion of masticatory muscles maintains a dominant temporalis (52.2%–68.1%), followed by the zygomasseteric complex (26.8%–43.9%) and medial pterygoid (3.8%–7.9%), but the relative importance of the temporalis and zygomasseteric complex varied across taxa (in an inverse relationship between them that was not observed in the medial pterygoid). The variation in the relative size of the temporalis and zygomasseteric complex essentially followed the gradient of diet composition in Didelphidae (Vieira & Astúa de Moraes, 2003) (Figure 6). *Lutreolina* and *Caluromys* generally are positioned at either end of the spectrum with diets with higher frequency of vertebrates and fruits, respectively. The temporalis was more dominant in *Lutreolina* (68.1%) and *Philander* (66.7%), with the lowest proportion of total muscle mass in *Caluromys* (52.2%). The pattern at the more carnivorous end was more similar to that observed in placental carnivores, but most genera still fall within the generalized form sensu Turnbull (1970), that is, temporalis accounting for over 50%, zygomasseteric complex for up to 35%, and pterygoid for less than 20% of total jaw‐closing musculature, except for the high proportion of the zygomasseteric complex in *Caluromys* (43.9%) and a low anatomical complexity of the jaw adductor muscle groups (Ercoli et al., 2023). *Marmosops*, *Marmosa*, and *Metachirus* had a typical range pattern for the more generalized members of the group (temporalis: 55%–60%, zygomasseteric complex: 27%–35%, pterygoid less than 12%).

A relatively larger temporalis increases the bite force at a wider gape (Thexton & Hiiemae, 1975). The well‐developed temporalis muscle mass in *Lutreolina* confirms the only previous anatomical description made by Delupi et al. (1997). The increased size of the temporalis mirrors the enlargement of the coronoid process of the mandible and temporal fossa in the cranium (Astúa & Guilhon, 2023). The sagittal crest also increases the area of origin of the superficial temporalis and is well‐developed in this genus, with a height that remains uniform along its length. This results in more vertically oriented fibers that provide a more effective lever arm for elevating the mandible. As in Delupi et al. (1997), we did not identify a zygomaticomandibular muscle in *Lutreolina*. It was not found in *Didelphis* either, but it is observed in most genera, even if with a degree of intertwining with the temporalis that has already been observed to some extent in other marsupials (Sharp & Trusler, 2015; Thomas et al., 2024). Yet, in *Didelphis*, we still detected fibers with slightly more vertical orientations than the deep masseter, similar to those of the zygomaticomandibular, just as described in *Didelphis virginiana* (Diogo et al., 2016). These fibers are absent in *Lutreolina*, which already show a more posterior orientation. The posterior orientation of the fibers in a relatively larger temporalis allows individuals to face anterior resistance in the mandible (Greaves, 1991). The capabilities of a greater bite force at a wider gape and resisting strong anteriorly directed forces on the mandible indicate an efficient apparatus for the predation of other vertebrates. The diet composition (Lessa et al., 2023; Vieira & Astúa de Moraes, 2003) and the morphology of the skull, with a short rostrum, a pronounced narrowing of the braincase, and wider coronoid process (Astúa de Moraes et al., 2000) also support *Lutreolina* as one of the most carnivorous genera of the Didelphidae. Nevertheless, the absence of the zygomaticomandibular in *Lutreolina* differs from strictly carnivorous marsupials, such as species of the genus *Dasyurus* (Thomas et al., 2024). The differentiation of a new muscle layer, such as the zygomaticomandibular, influences the arrangement of the fibers, which can increase the muscular strength of the zygomasseteric complex when there is a reduction in the length of the fascicles (Deeming et al., 2022). On the other hand, this can lead to different movements of the mandible (Astúa & Guilhon, 2023), and the absence of a complex with more muscles can prevent the muscle force from being dispersed to other lines of action.

The main topographical difference in the masseter group was observed in *Chironectes*, with the superficial masseter originating along the most ventral extension of the lateral face of the zygomatic arch, while it was restricted posteriorly in the maxilla, ventral or over the edge of the maxillojugal region in the other genera. The anatomy of the superficial masseter is similar to that of placental carnivores (Brassard, Merlin, Monchâtre‐Leroy, et al., 2020; Ito & Endo, 2016). The more vertical fibers of the superficial masseter in *Chironectes* result in a greater lever arm for jaw elevation. This anatomical difference from other genera may reflect a possibly more durophagic habit of the species, for crushing crustaceans and aquatic vertebrates that comprise a large proportion of its diet (Andrade, 2017; Astúa, 2015; Lessa et al., 2023). Unfortunately, we did not assess the anatomy of the temporalis and its ability to generate force or to exert bite force in *Chironectes* due to the condition of our only specimen. Although *Chironectes* is one of the few genera with any previous description of masticatory muscles (Sidebotham, 1885), we were unable to make a detailed anatomical comparison because of the lack of illustrations for a complete understanding of the description in that work.

A more developed zygomasseteric complex in *Caluromys* is coherent with ecomorphological studies that suggested a proportionally larger and mechanically advantageous masseter in *Caluromys* (Astúa de Moraes et al., 2000). A proportional increase in the medial pterygoid was also expected due to the enlargement of the insertion area on the angular process which, unlike other didelphids, is not medially inflexed (Sánchez‐Villagra & Smith, 1997). However, *Caluromys* had the relatively smallest medial pterygoid, even smaller than that of *Lutreolina* (Figure 6), with the internal arrangement influenced by the shape of the angular process. The insertion of the medial pterygoid in the other genera seems to result in fibers that insert closer to the angular process edge and on the support it provides along with the horizontal ramus of the mandible. The lack of inflection of the angular process resulted in a reduced medial pterygoid, with a less diverse fiber arrangement in the mediolateral plane, and inserting close to the edge of the angular process. The fiber path tended to be longer and resulted in relatively longer fascicles, as reflected by the highest residuals with cranium length. Longer fascicles reduce pPCSA (which was also indicated by the residuals) but allow for greater muscular extension of the medial pterygoid at the opening of the mandible. *Caluromys* is one of the genera that includes the greater proportion of fruits in its diet (Lessa et al., 2023; Vieira & Astúa de Moraes, 2003). A greater ability to open the mouth may facilitate fruit consumption at the molars (Herrel et al., 2024), while the highly developed complex zygomasseteric may contribute to producing compressive loads during biting, which is supported by the relatively higher corpus of the mandible at the level of the molars (Figueirido et al., 2013; Riley, 1985; Santana et al., 2022). The larger zygomasseteric complex associated with the relative reduction of the temporal resembles evolutionary paths followed by different mammal lineages towards herbivory (Ercoli et al., 2023).

The functional relationship between the anatomy of the masticatory muscles and diets with greater importance of vertebrates, in *Lutreolina* and *Chironectes*, and fruits, in *Caluromys*, indicates a greater facility in correlating form and function. Evolutionary analyses of functional morphology suggest the same pattern of unique selective regimes towards faunivory and herbivory, but not distinct for omnivorous taxa between the extremes (Grossnickle, 2020a). However, our data for diet showed a continuum between genera for the extremes different from Vieira and Astúa de Moraes (2003) and Lessa et al. (2023) (Figure 1). The diet composition data suggest a higher proportion of vertebrates in *Didelphis* and *Philander* and even a high proportion of fruit in *Lutreolina*. *Didelphis* is ranked in a more intermediate position between the genera in terms of temporalis size and *Lutreolina* has a decidedly less prominent masseter group than *Caluromys* (Figure 6). Therefore, we cannot determine that the anatomical diversification and muscle proportions of the adductor muscles are linked to the main variations in the diet, because they can represent the taxonomical differences, while the generalized pattern is already efficient for adding more vertebrates and/or fruits to the diet.

### 4.2 Bite force scaling

All the properties of the muscle groups (mass, average fascicle length, and pPCSA) and of the whole musculature, as well as bite force, increased isometrically with cranium length (Figures 7 and 8 and Table 2). Isometric relationships of the mass and total PCSA of the adductor musculature with indicators of body size have also been observed in other taxa (Hartstone‐Rose et al., 2022), especially in primates (Hartstone‐Rose et al., 2018; Perry, 2008; Perry & Wall, 2008), but bite force in carnivores is usually positively allometric (Hartstone‐Rose et al., 2022). Bite force is an important functional trait of the vertebrate masticatory apparatus that can determine an animal's ability to process the range of foods included in its diet (Deeming et al., 2022; Kraus et al., 2022; Mitchell et al., 2024). The isometric pattern detected in opossums indicates that the size and force produced by the adductor musculature, and delivered during the bite, are consistent with body size. Thus, *Didelphis*, as the living genus with the largest average body size among opossums (Astúa, 2015) and in our sample, can exert higher bite forces. Nonetheless, the bite force residuals of the *Didelphis* specimens were not high. This indicates that *Didelphis* can produce a higher absolute bite force, but not relatively high for its size. Only the bite force at the canine suggests moderate evidence for a relatively stronger bite force in the larger specimens. As muscle strength is isometric, the mechanical advantage of the canine bite seems to lead to a relative increase in bite force, probably associated with an improved robustness of the mandible (Silva‐Neto et al., 2024). In any case, the residuals show that *Didelphis* does not produce notably high bite forces for its skull size. *Philander*, on the other hand, can exert a high absolute and relative bite force on the first molar. *Marmosa* and *Caluromys* seem to have a masticatory apparatus that is more adapted to biting harder, as they had the highest residual bite force at the molar and canine. Inversely, the bite force residuals were lower for *Metachirus* at the molar, and *Marmosops* and *Monodelphis* at the canine (Figure 8).

The role of body size in determining features of the masticatory apparatus in opossums (as seen through isometric relationships) is likely directly related to the magnitude of morphological integration in the crania of Didelphidae. Shirai and Marroig (2010) showed that the skull of didelphids has high levels of morphological integration, as for all marsupials (Marroig et al., 2009; Porto et al., 2009), and with poorly distinct phenotypic modules in general. Thus, size variation is key to the functional responses of the skull in didelphids. In fact, mandible size variation has been proposed as key for enabling the group to diversify its diet (Silva‐Neto et al., 2024), and larger mandibular sizes have been linked to omnivorous opossum species, and differences in size between diet categories have also been noted in the group (Brum et al., 2023). Thus, size is one of the main factors structuring trophic relationships with bite force between didelphids, as a main factor in resource partitioning, as several opossum taxa co‐occur in small mammal communities, sharing the same environment with similar morphology and feeding on a similar diet. However, it is still unclear whether the increase in overall size of didelphids could be related to a possible selection on bite force for feeding, or conversely, the increase in bite force is a by‐product of overall size diversification, as size variation offers a faster and less resistant path to morphological diversification (i.e. a line of least evolutionary resistance) (Brum et al., 2023; Grossnickle, 2020a; Marroig et al., 2009; Mitchell et al., 2024). If diet were driven by body size range, it should have indicated size‐related tendencies and more specific dietary adaptations, as in primates (Marroig & Cheverud, 2005), which also demonstrate isometric adductor muscle size relationships. However, the general magnitude of skull integration in didelphids is associated with less flexibility in responding to selection than in primates, at least in the New World (Shirai & Marroig, 2010). Therefore, variation in body size or even skull size in didelphids may be influenced by other factors (Amador & Giannini, 2016; Grossnickle, 2020a), as regardless of the direction selection is pulling, the response tends to be associated with size (Astúa & Guilhon, 2023; Marroig et al., 2009; Shirai & Marroig, 2010). The consistent increase in bite force may have secondarily enabled the incorporation of other food items, due to the limitation of smaller species to prey on vertebrates or invertebrates with a hard exoskeleton (Astúa & Guilhon, 2023), even to meet the nutritional requirements of their larger size (Marroig & Cheverud, 2005).

### 4.3 Bite force, diet and diet mechanical challenge

We found no evidence of correlation of bite forces at the canine and first molar with diet or dietary mechanical challenge when corrected for size. Although the range of main items consumed varies considerably in the diet of didelphids, the lack of coupling between bite forces and diet variables seems to reflect the general similarity of their diets. We expected the impact of dietary mechanical challenge to be significant due to the importance of mechanical demands for food selection, chewing, and biting (Berthaume, 2016; Santana et al., 2022), but the correlation between estimated bite force and dietary mechanical resistance was also not observed in all the lineages (Hartstone‐Rose et al., 2022). However, *Didelphis* showed relatively higher indices of hard items in the diet (Figure 1) and had higher bite forces among the genera. *Chironectes* also presented a diet with a high dietary mechanical challenge. Even though it was not possible to obtain the bite force of *Chironectes* due to damage to the specimen, we can estimate a bite force on the molar consistently below that of *Didelphis* and *Philander* and above that of *Lutreolina*, based on the pPCSA of the zygomasseteric complex, which was more predictive of the bite force. If we consider the moment of force produced by the zygomasseteric complex, *Chironectes* has a bite force on the first molar only lower than that of *Didelphis*, due to the longer lever arm, which would be consistent with the higher level of dietary mechanical challenge for both genera. In contrast, *Marmosops* obtained the lowest dietary mechanical challenge index, but this was not reflected in the bite force, corrected for size or not.

### 4.4 Inferences for mastication

Opossums are considered morphologically conserved and functional models of primitive metatherians and therians (Amador & Giannini, 2016; Astúa & Guilhon, 2023; Diogo et al., 2016). Bhullar et al. (2019) described the ancestral tribosphenic chewing stroke of therian mammals based on the conservation of ancestral skull features in the short‐tailed opossum, *Monodelphis domestica*. According to their hypothesis, the simple and symmetrical pattern of eversion and inversion paired with the opening and closing of the mandible, respectively, and the roll of the mandible (i.e. rotation around the long axis of the hemimandible) are plesiomorphic characteristics of therian mastication. Although the hemimandibular rotation has already been described in *Didelphis* opossums (Crompton & Hiiemäe, 1970; Lieberman & Crompton, 2000), Bhullar et al. (2019, 2020) suggest that roll‐based processing is the principal mechanism of grinding in Cladotheria, contrary to Grossnickle's (2017) suggestion that the chewing cycles of the earliest cladotherians are yaw‐dominated (i.e., rotation about a dorso‐ventrally oriented axis). In opossums, these rotations help align the lower molar cusps with the upper molar cusps during occlusion and are possible due to the unfused symphysis which allows some independent movement between the hemimandibles (Davis et al., 2024; Lieberman & Crompton, 2000).

Stilson et al. (2023) described mandibular movements in *Didelphis virginiana* similar to those seen in *Monodelphis* during mastication (Bhullar et al., 2019), with a consistently greater magnitude of roll than yaw. These data may correspond to a general pattern in the masticatory stroke of opossums. The anatomy of the adductor muscles of the mandible is not very divergent in opossums in general, especially the muscles that participate in the roll (superficial masseter and medial pterygoid), which, associated with the relatively conserved morphology of the cranium (Chemisquy et al., 2021) and mandible (Silva‐Neto et al., 2024) may not lead to contrasting differences in the chewing of *Monodelphis* and *Didelphis*. Even the lateral component of the superficial masseter in *Chironectes*, which originates on the zygomatic arch, may contribute to the lateral sliding of the mandible (Ito & Endo, 2016). However, it is still unclear how the change in the morphology of the medial pterygoid in *Caluromys* relates to the movement of the mandible during mastication. The mechanical advantage for jaw rolling in *Caluromys* seems to be reduced with the lower complexity of the medial pterygoid in the mediolateral plane (Grossnickle, 2020b), while the smaller size of the medial pterygoid should contribute little to the movement of mandibular yaw (Grossnickle, 2020b). These results may bring the morphotype and mandibular movements of *Caluromys* closer to those of carnivores, with reduced transverse movement of the mandible (Ercoli et al., 2023; Grossnickle, 2020b). Therefore, *Caluromys* may have a simple orthal occlusion, which has also been proposed in the evolution of cladotherians (Grossnickle et al., 2022). This result strengthens the need to integrate other information, such as measurements of the lever arms in other planes for the yaw and roll movements of the mandible, muscle activation patterns, and hemimandibular kinematics of the mandible (Ercoli et al., 2023; Nabavizadeh, 2016; Stilson et al., 2023) to understand masticatory movements of *Caluromys*, as well as other opossums that remain poorly represented in the functional study of the masticatory apparatus.

## Supporting information


**Figure S1.** Photographs of adductor musculature dissection in Didelphis. Ts, m. temporalis pars superficialis; Tp, m. temporalis pars profunda; Ms, m. masseter pars superficialis; Mp, m. masseter pars profunda; Pm, m. pterygoideus medialis.

**Figure S2.** Photographs of adductor musculature dissection in Philander. Ts, m. temporalis pars superficialis; Tp, m. temporalis pars profunda; Ms, m. masseter pars superficialis; Mp, m. masseter pars profunda; Zg, m. zygomaticomandibularis; Pm, m. pterygoideus medialis.

**Figure S3.** Photographs of adductor musculature dissection in Caluromys. Ts, m. temporalis pars superficialis; Tp, m. temporalis pars profunda; Ms, m. masseter pars superficialis; Mp, m. masseter pars profunda; Zg, m. zygomaticomandibularis; Pm, m. pterygoideus medialis.

**Table S1.** Mass (in g), average fascicle length (FL, in cm), and pPCSA (in cm^2^) of the individual muscles of the specimens used in the study. See text for further explanations.
