# Supplementary figures and images for: Comparative and functional anatomy of masticatory muscles and bite force in opossums (Didelphimorphia, Didelphidae)
Source: Anat Rec (Hoboken). 2025 Apr 25;309(9):2346–67. doi: 10.1002/ar.25675 (PMC13432368; doi:10.1002/ar.25675)

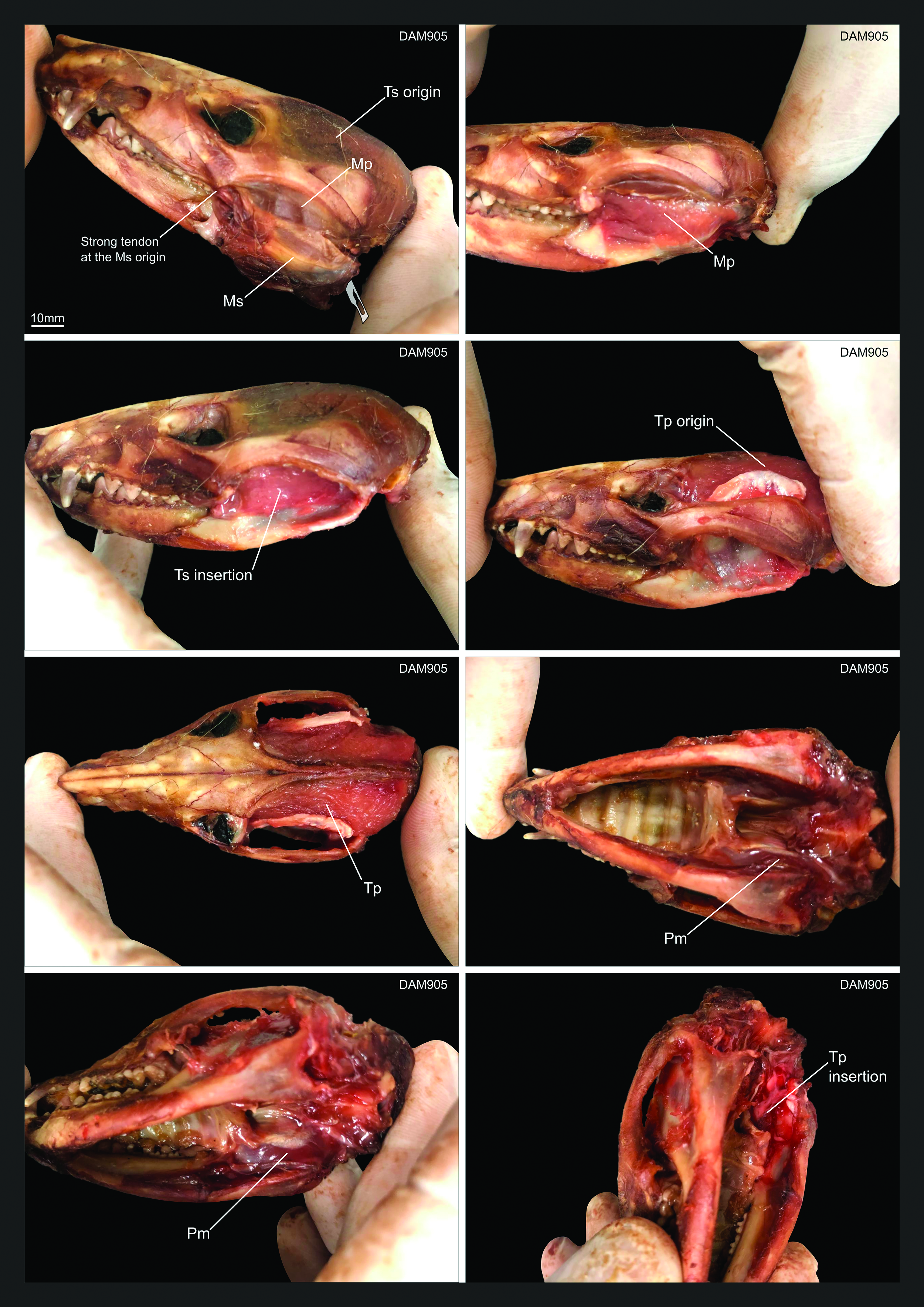

Supplement: Supplementary file 1 — Figure S1. Photographs of adductor musculature dissection in Didelphis. Ts, m. temporalis pars superficialis; Tp, m. temporalis pars profunda; Ms, m. masseter pars superficialis; Mp, m. masseter pars profunda; Pm, m. pterygoideus medialis. [file AR-309-2346-s003.tif]

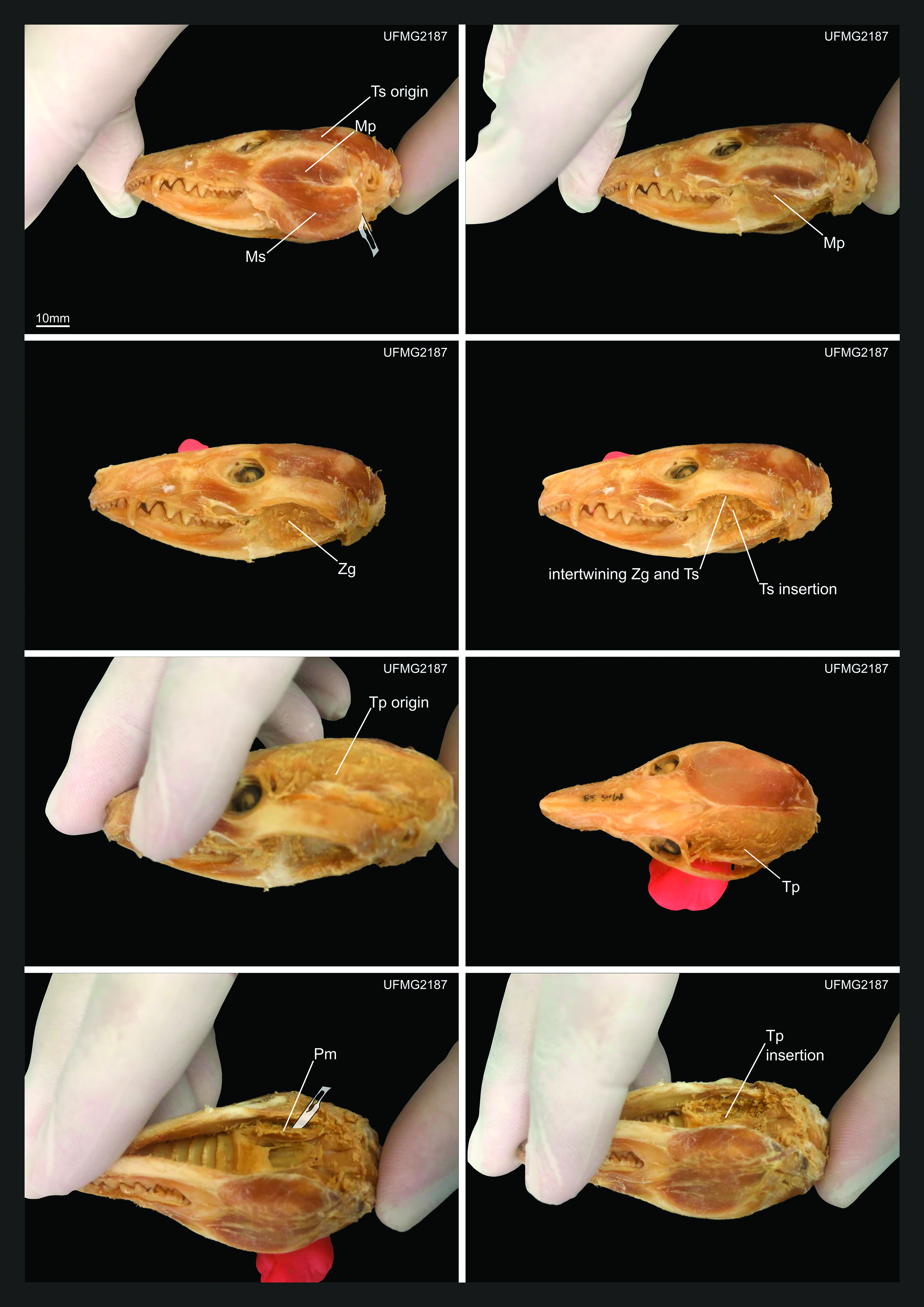

Supplement: Supplementary file 2 — Figure S2. Photographs of adductor musculature dissection in Philander. Ts, m. temporalis pars superficialis; Tp, m. temporalis pars profunda; Ms, m. masseter pars superficialis; Mp, m. masseter pars profunda; Zg, m. zygomaticomandibularis; Pm, m. pterygoideus medialis. [file AR-309-2346-s002.tif]

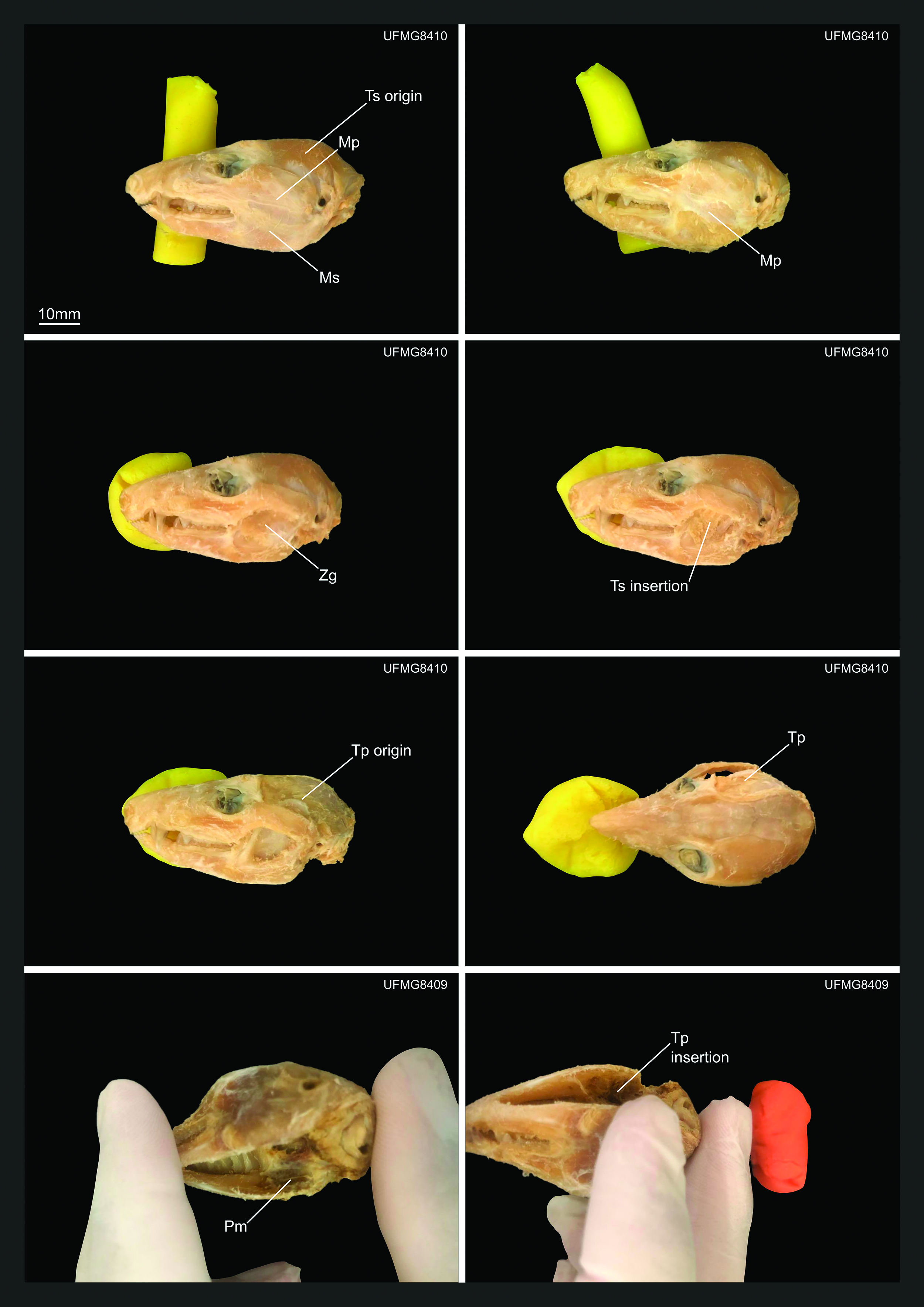

Supplement: Supplementary file 3 — Figure S3. Photographs of adductor musculature dissection in Caluromys. Ts, m. temporalis pars superficialis; Tp, m. temporalis pars profunda; Ms, m. masseter pars superficialis; Mp, m. masseter pars profunda; Zg, m. zygomaticomandibularis; Pm, m. pterygoideus medialis. [file AR-309-2346-s001.tif]
