# Supplementary material for: Comparative and functional anatomy of masticatory muscles and bite force in opossums (Didelphimorphia, Didelphidae)
Source: Anat Rec (Hoboken). 2025 Apr 25;309(9):2346–67. doi: 10.1002/ar.25675 (PMC13432368; doi:10.1002/ar.25675)
Supplement: Supplementary file 4 — Table S1. Mass (in g), average fascicle length (FL, in cm), and pPCSA (in cm2) of the individual muscles of the specimens used in the study. See text for further explanations. [file AR-309-2346-s004.docx]

Table S1. Mass (in g), average fascicle length (FL, in cm), and pPCSA (in cm²) of the individual muscles of the specimens used in the study. See text for further explanations.

| **ID** | **Superficial masseter** | | | **Deep masseter** | | | **Zygomaticomandibular** | | | **Superficial temporalis** | | | **Deep temporalis** | | | **Medial pterygoid** | | |
| --- | --- | --- | --- | --- | --- | --- | --- | --- | --- | --- | --- | --- | --- | --- | --- | --- | --- | --- |
|  | Mass | FL | pPCSA | Mass | FL | pPCSA | Mass | FL | pPCSA | Mass | FL | pPCSA | Mass | FL | pPCSA | Mass | FL | pPCSA |
| Did_alb_DAM904 | 1.280 | 1.372 | 0.989 | 0.510 | 0.829 | 0.652 |  |  |  | 2.640 | 1.145 | 2.444 | 0.960 | 0.912 | 1.115 | 0.370 | 0.386 | 1.015 |
| Did_alb_DAM905 | 1.200 | 1.116 | 1.140 | 0.420 | 0.909 | 0.490 |  |  |  | 2.470 | 1.050 | 2.494 | 1.010 | 0.915 | 1.170 | 0.410 | 0.511 | 0.850 |
| Did_alb_DAM903 | 2.840 | 1.738 | 1.732 | 1.000 | 1.224 | 0.866 |  |  |  | 5.000 | 1.340 | 3.956 | 3.150 | 1.197 | 2.789 | 0.550 | 0.648 | 0.900 |
| Phi_qui_UFMG2187 | 0.560 | 0.908 | 1.105 | 0.170 | 0.883 | 0.345 | 0.100 | 0.875 | 0.205 | 1.160 | 0.922 | 2.254 | 0.780 | 1.067 | 1.309 | 0.140 | 0.362 | 0.693 |
| Lut_cra_UFMG7991 | 0.170 | 0.634 | 0.480 | 0.040 | 0.590 | 0.121 |  |  |  | 0.400 | 0.944 | 0.759 | 0.280 | 0.697 | 0.720 | 0.040 | 0.273 | 0.263 |
| Lut_cra_UFMG7990 | 0.190 | 0.656 | 0.519 | 0.070 | 0.634 | 0.198 |  |  |  | 0.310 | 0.690 | 0.805 | 0.220 | 0.738 | 0.534 | 0.050 | 0.296 | 0.302 |
| Met_myo_UFMG8413 | 0.100 | 0.681 | 0.263 | 0.040 | 0.699 | 0.102 | 0.020 | 0.609 | 0.059 | 0.110 | 0.866 | 0.227 | 0.120 | 0.592 | 0.363 | 0.020 | 0.361 | 0.099 |
| Met_myo_UFMG2185 | 0.130 | 0.647 | 0.360 | 0.050 | 0.671 | 0.133 | 0.050 | 0.330 | 0.271 | 0.280 | 0.687 | 0.730 | 0.180 | 0.351 | 0.918 | 0.050 | 0.321 | 0.279 |
| Cal_UFMG8409 | 0.160 | 0.381 | 0.751 | 0.060 | 0.433 | 0.248 | 0.040 | 0.451 | 0.159 | 0.180 | 0.518 | 0.622 | 0.120 | 0.444 | 0.484 | 0.020 | 0.417 | 0.086 |
| Cal_UFMG8410 | 0.160 | 0.518 | 0.276 | 0.130 | 0.502 | 0.463 | 0.020 | 0.354 | 0.101 | 0.210 | 0.605 | 0.622 | 0.170 | 0.463 | 0.658 | 0.030 | 0.419 | 0.128 |
| Mar_UFMG8411 | 0.310 | 0.684 | 0.812 | 0.040 | 0.493 | 0.145 | 0.020 | 0.597 | 0.060 | 0.450 | 0.672 | 1.200 | 0.280 | 0.694 | 0.723 | 0.040 | 0.312 | 0.229 |
| Mar_mur_UFMG8412 | 0.040 | 0.414 | 0.173 | 0.010 | 0.358 | 0.050 | 0.008 | 0.334 | 0.044 | 0.060 | 0.457 | 0.235 | 0.030 | 0.343 | 0.157 | 0.014 | 0.180 | 0.144 |
| Mops_UFMG8406 | 0.090 | 0.460 | 0.350 | 0.020 | 0.330 | 0.109 | 0.010 | 0.323 | 0.055 | 0.160 | 0.544 | 0.527 | 0.070 | 0.475 | 0.264 | 0.030 | 0.250 | 0.215 |
| Mon_UFMG8403 | 0.040 | 0.564 | 0.127 | 0.020 | 0.476 | 0.075 | 0.020 | 0.265 | 0.135 | 0.080 | 0.566 | 0.253 | 0.080 | 0.518 | 0.276 | 0.010 | 0.236 | 0.076 |
| Chi_min_UFMG8408 | 0.150 | 0.920 | 0.292 | 0.100 | 0.812 | 0.221 | 0.150 | 0.742 | 0.362 |  |  |  |  |  |  | 0.110 | 0.370 | 0.533 |
